# Amyloids and the Heart: An Update

**DOI:** 10.3390/jcm13237210

**Published:** 2024-11-27

**Authors:** Filippos Triposkiadis, Alexandros Briasoulis, Andrew Xanthopoulos

**Affiliations:** 1 School of Medicine, European University Cyprus, 2404 Nicosia, Cyprus; 2 Department of Clinical Therapeutics, Faculty of Medicine, Alexandra Hospital, National and Kapodistrian University of Athens, 11528 Athens, Greece; alexbriasoulis@gmail.com; 3 Department of Cardiology, University Hospital of Larissa, 41110 Larissa, Greece; andrewvxanth@gmail.com

**Keywords:** cardiac amyloidosis, light chain, transthyretin, hereditary, wild type, treatment, comorbidities

## Abstract

Amyloids consist of fibrils that can be formed by a large variety of different precursor proteins. In localized amyloidosis, amyloids accumulate at the production site with a single organ being affected, whereas in systemic amyloidosis several organs are affected, with the heart being the most common, followed by the kidneys, liver, and the nervous system. The two most frequent systemic amyloidosis types affecting the heart in the vast majority (>95%) of cases are immunoglobulin light chain (AL) amyloidosis and transthyretin (TTR) amyloidosis (ATTR amyloidosis). Patients with amyloid cardiopathy (CA) often present with non-specific heart failure symptoms as well as other clinical manifestations depending on the organ or systems involved. However, there are some findings associated with amyloidosis called “red flags” (clinical, echocardiographic, magnetic resonance imaging), which may assist in guiding the physician to the correct diagnosis. The present state-of-the-art review summarizes the features of the various cardiac phenotypic expressions of amyloidosis, proposes a simplified pathway for its diagnosis, and highlights the rapidly evolving therapeutic landscape.

## 1. Introduction

**Figure 1:**
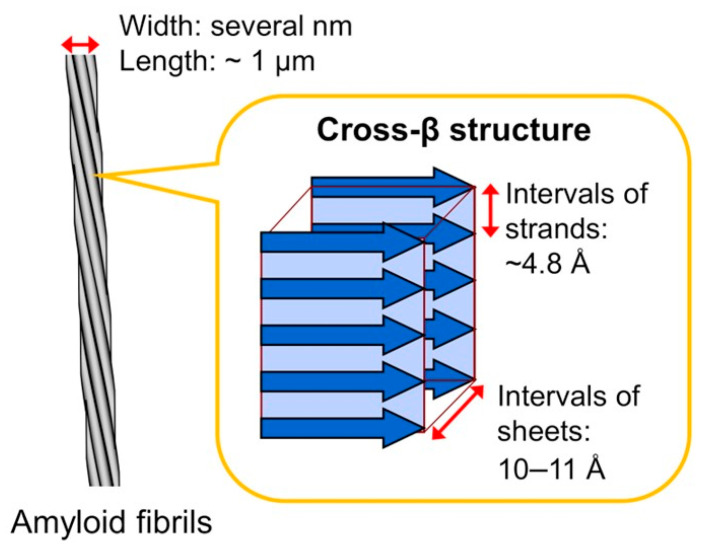
Schematic presentation of the structure of an amyloid fibril. Amyloid fibrils have needle-like and unbranched morphology consisting of laterally bundled protofilaments. Each protofilament has a cross-β structure, characterized by the presence of β-strands, which are stacked perpendicular to the fibril long axis. Adapted from Ref. [2].

Amyloids consist of fibrils that can be formed by a large variety of different precursor proteins and has been implicated in the pathology of a group of diseases known as the amyloidoses (Figure 1) [1,2]. More than 40 proteins are known to form insoluble amyloid fibrils, and the clinical manifestations of amyloidosis vary depending on the precursor protein [3]. Depending on the precursor protein, clinical manifestations in amyloidosis vary significantly. In localized amyloidoses, amyloid accumulates at the production site with only one organ being affected [4], whereas in systemic amyloidoses, several organs are affected, with the heart being the most common, followed by the kidneys, liver, and the nervous system [5]. The two most prevalent systemic amyloidosis types affecting the heart in the vast majority (>95%) of cases are immunoglobulin light chain (AL) amyloidosis [6] and transthyretin (TTR) amyloidosis (ATTR amyloidosis) [7]. Amyloidoses rarely associated with cardiac involvement are AA amyloidosis occurring in the setting of systemic inflammatory processes such as autoimmune disorders or chronic infections as well as with malignant neoplastic disorders [8] and in dialysis-related amyloidosis [9]. The diagnosis of amyloidosis and specifically of cardiac involvement is often fortuitous on imaging or a tissue biopsy necessitating a call for a proactive action focusing on early disease recognition in view of the currently available treatments for ATTR amyloidosis cardiomyopathy. Deficient awareness, similarity of symptoms with those brought about by more common disorders, and absence of a single non-invasive diagnostic gold standard contribute to the deferred diagnosis. This descriptive review attempts to represent the state of the art of amyloid cardiopathy (CA) by summarizing the features of the various cardiac phenotypic expressions of amyloidosis and highlight the rapidly evolving relevant therapeutic landscape.

## 2. Types of Amyloid Cardiopathy

### 2.1. AL Amyloidosis Cardiopathy (AL-CA)

AL amyloidosis is the most common form of systemic amyloidosis with estimated incidence and 20-year prevalence rates of 10 and 51 cases per million individuals, respectively [10]. AL results from clonal expansion of differentiated plasma cells producing immunoglobulin free light chains (FLCs), which are prone to misfolding and secreted in excess compared with heavy chains [11,12]. There are two types of light chains, κ and λ, and each consist of a variable N terminal Ig domain attached to a constant C-terminal Ig domain. While excess FLC production is observed in several plasma cell disorders (e.g., monoclonal gammopathy of undetermined significance, multiple myeloma, and Waldenstrom macroglobulinemia), only a small part of FLCs can produce amyloid deposits in vivo. The λ light chains are almost twice as common as κ in systemic AL amyloidosis [13]. DNA sequencing studies have demonstrated germline gene mutations on the variable λ region that induce thermodynamic instability of the protein, are strongly associated with amyloidosis development, and are accountable for the tendency of λ light chains to generate amyloid deposits [14]. Light chains that are amyloidogenic exhibit reduced kinetical stability and susceptibility to endoproteolysis, which result in the release of amyloidogenic light chain fragments prone to inappropriate aggregation.

Amyloid infiltration of the heart is present in ≈80% of cases and adversely affects morbidity and mortality [15]. AL-CA bestows a poorer prognosis than ATTR-CA, despite the fact that the cardiac amyloid burden and left ventricular (LV) mass in ATTR-CA are usually greater than those of AL-CA [16]. Infiltrations with AL amyloids are present in all cardiac chambers and lead to biventricular myocardial wall thickening, restrictive cardiomyopathy, and HF, which are frequently associated with the presence of atrial fibrillation and conduction abnormalities resulting in bradyarrhythmia [17]. Studies evaluating myocardial composition in AL-CA reported that myocardial extracellular volume (ECV) was expanded both in active AL-CA and remission AL-CA, whereas myocardial T2 relaxation times as well as native T1 times were higher in the remission than the active AL-CA group, indicating a divergent myocardial composition in these two settings [18]. As AL amyloids can infiltrate almost any organ in the body, extra-cardiac manifestations are common and often result in non-specific disease presentation rendering the diagnosis cumbersome and often delayed [19]. Extra-cardiac manifestations are common since AL amyloid can infiltrate almost any organ in the body, resulting in a heterogeneous and often non-specific disease presentation rendering diagnosis cumbersome and often delayed. However, almost one third of the patients have specific clinical signs such as macroglossia and peri-orbital bruising [20]. Other clinical manifestations of AL-CA include dyspnea, weight loss, and fatigue. Dyspnea, which usually progresses rapidly, frequently co-exists with peripheral edema and/or ascites. The latter is not only due to right heart failure [21] but also to renal amyloid infiltration resulting in heavy proteinuria and hypoalbuminemia [22]. Cardiac symptoms 101 usually appear in advanced cardiac involvement.

### 2.2. ATTR Amyloidosis Cardiopathy (ATTR-CA)

TTR, which is synthesized predominantly in the liver, is predominantly a serum transporter for holo-retinol-binding protein and less so a thyroxine transport protein, as <1% of transthyretin in plasma is thyroxine-bound [23]. TTR is also produced by the choroid plexus and released into the cerebrospinal fluid, in which it serves as the primary thyroxine carrier [24]. TTR comprises 4 identical subunits rich in β-sheets assembling into two αβ dimers, 109 which contact each other at an extended β-sheet forming two hormone-binding sites, and 110 where thyroxine binds [24,25]. A prerequisite for TTR amyloid fibril formation is the dissociation of the tetramer into monomers, which subsequently assemble to generate insoluble amyloid fibrils [26,27]. Amyloid fibril formation in ATTR amyloidosis results either from an aging-linked process (wild-type ATTR amyloidosis; ATTRwt) being present in approximately 10% to 15% of older adults with HF or from a destabilizing mutation (hereditary ATTR amyloidosis; ATTRv amyloidosis) [28,29]. Physiological fibrinolysis has been proposed to significantly contribute to TTR amyloid generation in vivo. It seems that under physiological conditions in vitro, plasmin selectively cleaves TTR between the 48 and 49 residues, resulting in a release of truncated and full-length oligomers, which aggregate rapidly forming fibrils [30].

#### 2.2.1. Cardiopathy in ATTR Amyloidosis (ATTR-CA)

ATTR-CA is a major cause of mortality in ATTR amyloidosis, with varying survival rates depending on the severity of cardiac involvement. The exact epidemiology of ATTR-CA has not been outlined, and a true estimate of its prevalence is difficult. At autopsy, ATTR-CA is typically [31] present in roughly 25% of elderly people aged ≥85 years [32], and for most decedents with amyloidosis, the disease was not diagnosed prior to their death. In a study conducted in the Nordic countries, the prevalence of ATTR-CA per 100,000 inhabitants in 2018 ranged from 1.4 to 5.0 and was followed by a steep increase over time [33]. In the same study, the median survival from diagnosis was 30 months for ATTR-CA patients and 67 months for matched HF patients, and survival was significantly lower for female than for male ATTR-CA patients (median survival: 22 and 36 months), whereas no significant difference was observed in the HF cohort [33].

ATTR-CA includes a wide range of presentations, the classic being restrictive cardiomyopathy and HF [34]. Although HF in the setting of ATTR-CA has been linked with HF and preserved LVEF, recent compelling evidence strongly suggests that HF may occur at any LVEF level [35,36]. It is likely that patients with ATTR-CA at the lower end of the HF spectrum are diagnosed later in the disease course and perhaps with a more “burned out” physiology, as suggested by a relatively high proportion of reduced LVEF with remodeled ventricles. Atrial amyloidosis, which has been attributed to hemodynamic effects of mechanical overload secondary to ventricular diastolic dysfunction, atrial amyloid infiltration, and amyloid-induced damage of atrial cardiomyocytes, is considered an early manifestation of ATTR-CA and increases the risk for atrial fibrillation and thromboembolic events [37]. Occasionally, atrial amyloidosis may be present even without systemic disease and ventricular involvement (isolated atrial amyloidosis) due to local overproduction of atrial natriuretic peptide (ANP) [37]. Finally, amyloid infiltration in the aortic valve can develop and/or worsen stenosis, and as a result ATTR-CA often co-exists with aortic stenosis in the elderly with the combination of the two disorders worsening the prognosis [38].

ATTR amyloidosis CA severely worsens the quality of life (QoL) of the affected patients. As QoL is a subjective term, which varies depending on several diverse factors and differs from individual to individual, two 30-item questionnaires were tested in the ITALY study for ATTRwt and ATTRv CA, with relevant scores ranging from 100 (best) to 0 (worst) [39]. The ITALY questionnaires, the first specific patient-reported outcome measures, proved feasible to complete and strongly related to non-ATTR-specific measures of QoL.

#### 2.2.2. Specific Features and Comorbidities of ATTR-CA

As ATTR-CA develops in the setting of both ATTRwt and ATTRv amyloidoses, which are systemic diseases with differing features, the clinical recognition of ATTR-CA is difficult, as it is distorted by the co-existing conditions and morbidities.

ATTRwt amyloidosis has classically been considered a cardiomyopathy of the elderly, particularly males. In this regard, autopsy studies have revealed that many elderly individuals have TTRwt depositions in the heart, including those without a history of underlying disease [40,41]. In addition to ATTR-CA, however, ATTRwt amyloidosis may present features indicative of carpal tunnel syndrome, which often pre-exist ATTR-CA [42]. Conversely, analysis of tenosynovial tissue specimens obtained during carpal tunnel surgery revealed that many patients diagnosed with “idiopathic” carpal tunnel syndrome had TTRwt amyloid deposits [43]. Further, an association between TTRwt deposition in ligaments and spinal canal stenosis [44,45] has been reported, suggesting a TTR tendency to deposit in organs vulnerable to shear stress [46]. Finally, myopathy may be the initial manifestation of ATTRwt, expanding the ATTRwt phenotypic spectrum and necessitating increased physicians awareness of this disease during differential diagnosis of myopathy [47].

ATTRv amyloidosis is characterized by genotypic and phenotypic heterogeneity. More than 150 TTR gene mutations have been reported, but only a minority has been implicated in ATTRv amyloidosis pathogenesis [48]. Most causative ATTRv amyloidosis mutations are gain-of-function ones with autosomal dominant inheritance, albeit with highly variable penetrance and expressivity, even within the same family [49]. In the Transthyretin Amyloidosis Outcomes Survey (THAOS) registry, over 30 different pathogenic TTR variants were reported, the most common being Val122Ile (45%), Thr60Ala (20%), and Val30Met (6%) [50]. The V122I variant is reported to be present in 3–4% of 187 Black Americans, but improvements in disease awareness most likely will lead to a 188 increase in diagnosis rate in these individuals [51]. In ATTRv, amyloid fibrils deposit in the endoneurium of peripheral nerves, leading to atrophy of Schwann cells close to amyloid fibrils and blood–nerve barrier disruption [52].

It should be noted that the true penetrance for amyloid mutations has largely not been established pathologically, and the mutation penetrance reported depends on the diagnostic modalities used to document its presence [53,54]. A consequence of the genetic heterogeneity observed in ATTRv amyloidosis is that it is difficult for a “typical presentation” to be defined, as presentation is variable [55]. Amyloid neuropathy is common and often associated with generalized autonomic failure. Less common presentations of ATTRv include bulbar complications, ocular manifestations, urinary bladder dysfunction, renal disease, and myopathy [55].

#### 2.2.3. Amyloidoses Rarely Affecting the Heart

AA amyloidosis is associated with autoinflammatory conditions such as rheumatoid arthritis, inflammatory bowel disease, and hidradenitis suppurativa, particularly when the diagnosis is delayed. SAA (serum amyloid-associated protein) is elevated, and cardiac involvement in AA amyloidosis is always preceded by renal involvement. Effective control of the underlying inflammatory process can halt disease progression and even reverse organ damage [56].

β2-microglobulin is the precursor protein for CA associated with long-term dialysis. CA related to β2-microglobulin would occur with low-flow dialysis membranes in patients on dialysis for >9 years in duration. However, newer dialysis technologies reduce serum β2-microglobulin levels in chronic dialysis patients and appear to reduce the risk of developing this form of systemic amyloidosis [57].

## 3. Diagnosis of Amyloid Cardiopathy

### 3.1. “Red Flags”

**Figure 2:**
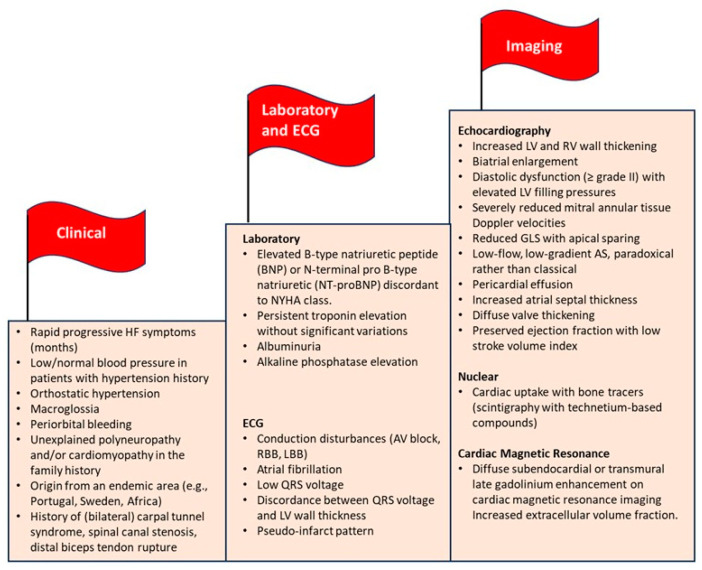
“Red flags” for amyloidosis with cardiac involvement. ECG, electrocardiogram; NYHA, New York Class Association; ECG, electrocardiogram; RBB, right bundle branch block; LBBB, left bundle branch block; LV, left ventricular; RV, right ventricular; GLS, global longitudinal strain; AS, aortic stenosis. Figure based on data from Refs. [58,59,60].

Patients with amyloid CA often present with non-specific HF symptoms as well as other clinical manifestations depending on the organ or systems involved. However, there are some findings associated with amyloidosis called “red flags”, which may be helpful in guiding the physician to the correct diagnosis [58,59,60] (Figure 2). Therefore, a high level of suspicion is of utmost importance, since cardiac amyloidosis is not rare, especially in the elderly [61]. Some of the red flags, like hypotension or normal blood pressure in previously hypertensive patients or deteriorating intolerance to standard HF or anti-hypertensive medications, may occur in every disease form, whereas others are specific for certain types of amyloidosis. For example, macroglossia [62] and peri-orbital bleedings [63] are extra-cardiac manifestations pathognomonic of systemic AL and are observed in 10–15% of patients. The history of ATTR amyloidosis patients often includes orthopedic conditions including carpal tunnel syndrome, spinal canal stenosis, or distal biceps tendon rupture, which in many patients appear years before the onset of HF symptoms [64,65,66]. Finally, other red flags include unexplained polyneuropathy, which may develop in ATTRv amyloidosis with a mixed phenotype or AL, and a family history of unexplained cardiomyopathy and/or polyneuropathy may be indicative of ATTRv disease origin from specific endemic area [67]. Finally, AL amyloidosis is a rapidly progressive disease, whereas ATTR amyloidosis progression is significantly slower.

### 3.2. Laboratory Testing

The initial step when amyloidosis is suspected is determining whether monoclonal free light chains are present or not in the serum or urine [68]. The most efficient and effective tests to exclude AL are immunofixation electrophoresis (IFE) of the serum and urine, and the sFLC assay, which measures circulating k and l free light chains and the k/l ratio. If there is no monoclonal protein on IFE of the serum and urine and the k/l ratio is within normal limits adjusted for renal function, AL can be virtually excluded (23,24). In cases with pathologic results, referral to a specialized amyloidosis center is mandatory, as a definite AL amyloidosis diagnosis needs to be biopsy proven [69,70]. The levels of cardiac biomarkers NT-proBNP and troponin should be measured, as both of them are highly sensitive, albeit not specific [71,72], but are important for risk stratification in established amyloidosis CA [73,74]. Albuminuria or alkaline phosphatase should also be determined in AL amyloidosis due to the frequent involvement of kidneys and liver.

### 3.3. Electrocardiography

Electrocardiographic (ECG) abnormalities such as low-voltage QRS and a poor R-wave progression in precordial leads are important findings, whereas both ventricular and supraventricular arrhythmias and conduction disturbances are common [75].

### 3.4. Echocardiography

**Figure 3:**
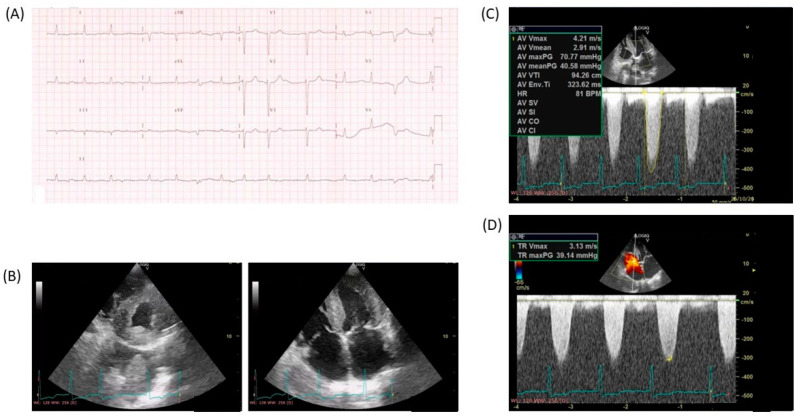
Eighty-one-year-old female patients with a history of diabetes and chronic kidney disease complaining of gradually increasing dyspnea (New York Heart Association II–III) and lower limb edema in the last 3 months. Heart blood pressure at admission was 110/85 mmHg and SpO2: 95%. The electrocardiogram (**A**) shows atrial fibrillation and low QRS voltage, whereas the echocardiogram (**B**) shows severe thickening of the left ventricular wall and the interventricular septum in the short axis view (**left**) and the four-chamber view (**right**). Continuous Doppler interrogation across the aortic valve (**C**) shows severe aortic stenosis with a maximal pressure gradient ≈ 71 mmHg and a mean pressure gradient ≈ 41 mmHg. (**D**) Continuous Doppler interrogation across the tricuspid valve shows tricuspid regurgitation with an abnormal maximal regurgitant jet velocity of 3.1 m/s, suggestive of pulmonary hypertension.

Echocardiography (ECHO) is the most widely used imaging modality for the diagnosis of amyloidosis CA. Although echocardiography is insufficient by itself to diagnose and specify the type of CA (ATTR amyloidosis vs. AL amyloidosis), it is a fundamental part of the diagnostic, prognostic, and ongoing management of amyloid CA patients [60]. The most common finding is LV wall thickening, which is not due to LV hypertrophy (LVH) but extracellular amyloid depositions, and it is frequently associated with low QRS voltage (Figure 3). In the absence of other causes of LVH/LV wall thickening (arterial hypertension, hypertrophic cardiomyopathy, aortic stenosis, etc.), amyloidosis should be suspected, although a normal thickness, especially in female patients in the early disease stages, does exclude amyloid CA. Thickening of the right ventricle, the interatrial septum, and the valves are a frequent finding [76,77]. “Granular sparkling”, a traditional sign characterized by increased echogenicity of the myocardial walls, may be observed in up to 25% of the patients, but it is not specific as it may appear in other diseases such as myocarditis [78]. Pericardial effusion is frequently observed, especially in AL.

Three indicators particularly useful for the evaluation LV function with echocardiography are the mitral flow pattern, LV strain, and LV stroke volume [79]. LVEF is usually preserved in early amyloid CA, but it may decline as disease progresses, whereas both longitudinal and diastolic dysfunction can be detected in the early disease stages [60]. Severe diastolic dysfunction accompanied by elevated ventricular filling pressures is a sign of severe disease stage and frequently co-exists with biatrial enlargement [80]. Reduced longitudinal strain assessed by speckle tracking echocardiography (STE) in basal and midventricular segments with preserved longitudinal strain in the apical segments is referred to as “apical sparing”, and it is a specific pattern distinguishing amyloidosis from other forms of thickening/hypertrophy. Nevertheless, apical sparing is observed in approximately one third of amyloid CA patients [81], and it may also be observed in other diseases (Fabry disease, severe chronic kidney disease, etc.) [82]. Finally, echocardiography, besides demonstrating the above-mentioned as typical, morphological, and functional, renders the evaluation of left ventricular function with novel indices feasible, such as the pressure–strain loop-derived global myocardial work index (GWI), which is impaired in amyloid cardiomyopathy [83,84,85].

Left atrial function assessed by speckle tracking echocardiography is significantly impaired in amyloidosis CA patients compared with those affected by hypertrophic cardiomyopathy and healthy controls, highlighting the potential supportive role of STE in the early detection and management of amyloid CA [86]. Several echocardiographic variables reflecting specific morphological and functional features of amyloid CA have been used to develop multiparametric scores in order to establish the diagnosis of CA in patients with light chain (AL) amyloidosis or those with increased heart wall thickness with suspected cardiac amyloid infiltration [87].

### 3.5. Cardiac Magnetic Resonance Imaging (CMRI)

CMRI plays a vital role in the diagnosis of cardiac amyloidosis, as besides the evaluation of cardiac morphology and function, it renders myocardial tissue characterization feasible by providing additional information about the myocardium, including inflammation, infiltration, and fibrosis [88]. Following the administration of gadolinium, the appearance and pattern of late gadolinium enhancement (LGE) are specific to different diseases. Amyloid CA is usually characterized by global subendocardial LGE in the early stages and global transmural LGE in the advanced disease stages. Besides diagnosis of amyloid CA, the LGE provides prognostic information [89]. Further, T1-mapping techniques of CMRI, despite some technical limitations, may be used for the estimation of extracellular space expansion owing to fibrosis, edema, or amyloid deposition [90]. Quantification of amyloid burden becomes feasible by employing contrast-enhanced T1-mapping, which allows calculation of the extracellular volume [91]. Importantly, CMRI can contribute to the differentiation between amyloid cardiomyopathy and other diseases characterized by ventricular wall thickening, such as hypertrophic cardiomyopathy, Anderson–Fabry disease, and mitochondrial cardiomyopathies [92].

Novel promising CMRI techniques include diffusion tensor imaging (DTI), which may provide a means for evaluating microstructural abnormalities in amyloid CA [93], and magnetic resonance spectroscopy which determines myocardial triglyceride level, which may be significantly decreased in amyloid CA [94,95].

### 3.6. Cardiac Scintigraphy

**Figure 4:**
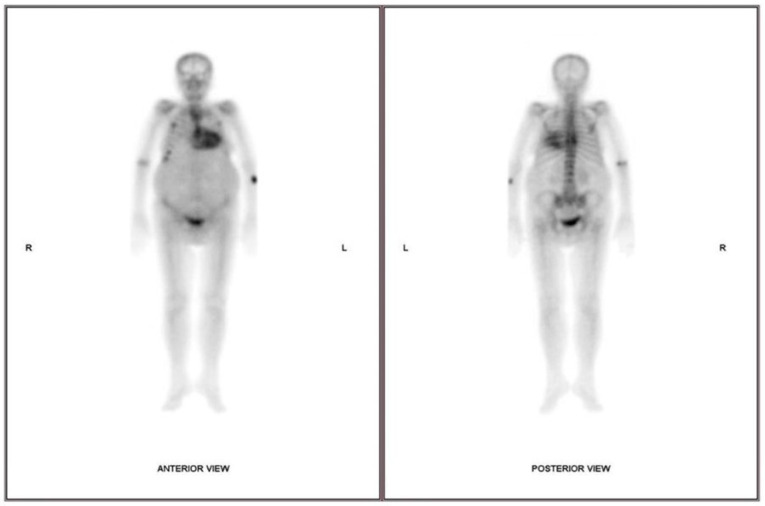
The 99m Tc-pyrophosphate scintigraphy of the patient above is indicative of transthyretin amyloidosis with a Perugini score = 3 (cardiac uptake > rib uptake).

Cardiac scintigraphy, through the use of technetium-labeled radioactive tracers (^99m^Tcpyrophosphate [99mTc-PYP], ^99m^Tc-3,3-diphosphono 1,2-propanodicarboxylic acid [99mTc-DPD], and 99^m^Tc-hydroxymethylene diphosphonate [99mTc HMDP]), has been increasingly employed for the diagnosis of ATTR-CA [96]. Although the underlying imaging mechanism has not been entirely delineated, there is evidence to suggest that microcalcifications present in the amyloid deposits and calcium-binding domains within the amyloid fibrils contribute to this process [97]. Radiotracer accumulation is significantly higher in ATTR-CA compared with AL-CA, and this may be due to the fact that binding of radiotracers to amyloid-containing hearts depends on the irregular presence of clouds of very tiny calcifications in ATTR amyloidosis which seem to be not directly related to amyloid fibrils [98]. The Peruggini grading scale is a semi-quantitative method of cardiac scoring 99mTc-DPD, 99mTc-Pyrophosphate, or 99mTc-HMDP scintigraphy in the investigation of amyloid CA (particularly ATTR-CA). The Peruggini scale uses visual comparison of tracer uptake between the myocardium and the ribs. In this regard, grade 0 is characterized by no cardiac uptake with normal rib uptake; grade 1 by cardiac uptake of less than rib uptake; grade 2 by cardiac uptake equal to rib uptake; grade 3 by cardiac uptake exceeding rib uptake with mild or absent rib uptake [99]. Visual scores of 2 or more planar are interpreted as ATTR-positive studies, whereas those less than 2 are considered ATTR-negative (Figure 4) [100]. It should be noted, however, that cardiac scintigraphy with ^99m^Tc radioactive tracers is unsuitable for the evaluation of AL, does not provide true quantitative assessment of amyloid burden, and does not allow monitoring of therapy response, and its sensitivity is suboptimal for the early detection of amyloid CA [101]. As the visual grading approach has limitations, especially for planar imaging, semi-quantitative parameters, such as the heart to contralateral lung (HCL) ratio and the heart to whole body ratio [102,103], have been increasingly used, increasing diagnostic accuracy and improving risk stratification [104]. However, these methods have limitations, as they are not suitable for early diagnosis, selection of the appropriate candidates for therapy, and monitoring of treatment [100]. SPECT/CT imaging is currently preferred compared with planar or SPECT-only scanning, due to its enhanced diagnostic accuracy, smaller inter- and intraobserver variability, and reduced false-positive rates [105].

Finally, it has been recently proposed that the combined analysis of ECG, echocardiographic, and scintigraphic data significantly increases the probability of diagnosis and differentiation of ATTR amyloidosis [106].

### 3.7. Positron Emission Tomography (PET)

Several radioactive, amyloid-targeting PET tracers have been used for the detection of amyloidosis CA. In an early report using ^11^C-PIB, patients with AL- CA had increased myocardial uptake [107], whereas another PET study using ^18^F florbetapir AL-CA showed significantly higher uptake than ATTR-CA [108]. Despite the presence of a considerable uptake overlap between AL-CA and ATTR-CA on amyloid-targeting PET, there is evidence to suggest that there is a higher radioactive tracer uptake in AL-CA compared with ATTR-CA [109], suggesting that cardiac scintigraphy and amyloid-424-targeting PET may complement each other. Further, myocardial uptake on ^11^C PIB PET in AL has a prognostic value as demonstrated in a recent study, in which patients with AL-CA with a higher ^11^C PIB PET uptake exhibited a greater risk for HF and death than those with a lower uptake [110]. One of the key recent developments in PET relevant to amyloidosis CA is quantitative imaging, which compared with visual assessment offers a more objective and reproducible measurement of myocardial uptake and may substantially improve early amyloidosis diagnosis, risk stratification, and follow-up assessments in amyloidosis CA [111,112,113].

A simplified pathway for the diagnosis of cardiac amyloidosis is presented in Table 1.

### 3.8. Limitations of Amyloidosis Imaging

Clinicopathological correlations with advanced imaging modalities provide an understanding of the significance of specific imaging findings in various diseases, including acute myocardial infarction with reperfusion injury and microvascular obstruction [114], inflammatory diseases [115], and primary and secondary cardiomyopathies [116,117,118]. A disease where autopsy studies can improve the accuracy of imaging modalities is cardiac amyloidosis. A recent meta-analysis reported that CMRI detects cardiac amyloidosis with sensitivity and specificity values of approximately 85–90% despite the lack of amyloidosis confirmation using autopsy as the gold standard [119]. The use of endo-myocardial biopsy instead of autopsy findings is less accurate regarding the evaluation of imaging findings. Indeed, assessing ATTR-CA with technetium-labelled cardiac scintigraphy has been reported to have a sensitivity of 99% and a specificity of 86%, with false positives largely deriving from the presence of AL [120]. Nevertheless, there has not been autopsy confirmation of these high sensitivities and specificities. Besides the considerable bias introduced by the fact that subjects were largely selected on the basis of a high likelihood of ATTR-CA [103], the phosphate-containing radioactive tracers used in cardiac scintigraphy are not TTR-specific and may be bound to myocardial microcalcifications in the myocardium [97], which are present in several pathologies [97,121,122]. This drawback is highlighted by the significant difference in ATTRwt amyloidosis whole-population incidence between autopsy and imaging studies in the elderly. At autopsy, ATTR-CA is present in approximately 25% of elderly people ≥ 85 years [32] and for most decedents with systemic amyloidosis, whereas through conventional bone scanning ATTR-CA was only detected in about 11% of patients ≥ 85 years [123]. Studies combining cardiac imaging and autopsy could clarify the causes of this disparity. Pathologically, amyloids can involve the heart with extensive interstitial deposits, and by occlusive vascular deposition they can involve the small intra-myocardial (penetrating) vessels [53,124,125]. Through small vessels obstruction, cardiac amyloidosis may lead to an initial presentation similar to coronary artery disease, and it is doubtful whether current imaging modalities can detect amyloid-induced intra-myocardial vascular involvement.

## 4. Treatment of Amyloid Cardiopathy

**Figure 5:**
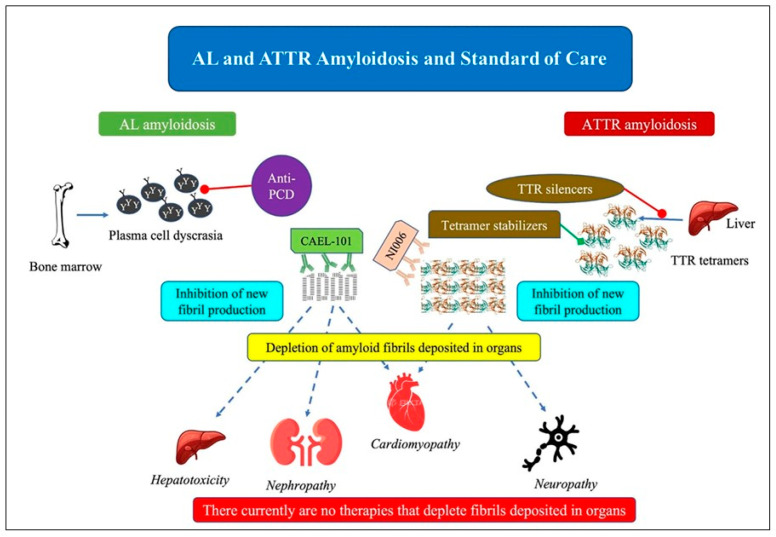
Current treatment of light chain amyloidosis (AL amyloidosis; anti PCD) and transthyretin (TTR) amyloidosis (ATTR amyloidosis; TTR silencers, tetramer stabilizers). Disease manifestations are in italics. Identification of the amyloidosis type and fibril characterization are crucial prior to treatment initiation since treatments for AL amyloidosis and ATTR amyloidosis differ significantly. For both AL amyloidosis and ATTR amyloidosis, the target treatment is inhibition of further generation and myocardial deposition of amyloid fibrils in combination with supportive care. Adapted from Ref. [126].

As treatments for AL amyloidosis and ATTR amyloidosis significantly differ, identification of the amyloidosis type and fibril characterization are crucial prior to treatment initiation (Figure 5) [126]. Treatment of amyloid CA is based on assessment of disease burden and patient risk stratification. For both AL amyloidosis and ATTR amyloidosis, the target of the standard of care is the prevention of further synthesis and deposition of amyloid fibrils in combination with supportive care. Treatment of amyloid CA includes specific treatment targeting the cause of amyloidogenesis and non-specific treatment of HF, conduction disturbances, and arrhythmias.

### 4.1. Specific Treatment of AL-CA

Management of AL-CA requires referral to specialized centers [127]. Stagings based on cardiac and renal biomarkers guide the treatment choice. The combination of daratumumab, cyclophosphamide, bortezomib, and dexamethasone (dara-CyBorD) is the current standard of care, whereas autologous stem cell transplantation is performed in selected patients, namely those with unsatisfactory response to dara-CyBorD [128]. Preliminary, encouraging findings suggest that the clearance of amyloid deposits may be enhanced by specific antibodies, which target amyloid fibrils [129]. Admittedly, evaluating the therapeutic effect of the anti-amyloid agents remains an unresolved research issue. A favorable early and sustained hematologic response is the treatment goal. An AL amyloidosis relapse can be managed with daratumumab combination, but novel more effective regimens are needed in this setting [127]. Immunomodulatory agents are currently the basis of rescue therapy, whereas targeting B-cell maturation antigen with immunomodulatory agents and Bcl-2 inhibitors are promising alternatives [130].

### 4.2. Specific Treatment of ATTR-CA

Therapies targeting different steps in TTR production provide new alternatives for treating patients suffering from this devastating disease.

#### 4.2.1. Interventions Targeting TTR Synthesis in the Liver

Proposed causal treatment modalities in TTR amyloidosis include blocking TTR synthesis in the liver using small interfering RNA (siRNA) or antisense oligonucleotide (ASO) technologies, stabilizing TTR tetramers or disrupting TTR fibrils [131].

The planned and pinpoint modification of the host genome using engineered nucleases (gene editing) is a seminal advancement in modern medicine, as this technique has the potential to cure genetic disorders stemming from mutations in a single gene such as ATTRv [132]. Gene editing has been revolutionized by recent technological improvements, particularly Clustered Regularly Interspaced Short Palindromic Repeats (CRISPR)/CRISPR associated nuclease 9 (Cas9)-based technology (CRISPR-Cas9), which enables in vivo modification of the cellular genome for therapeutic purposes [133].

**Figure 6:**
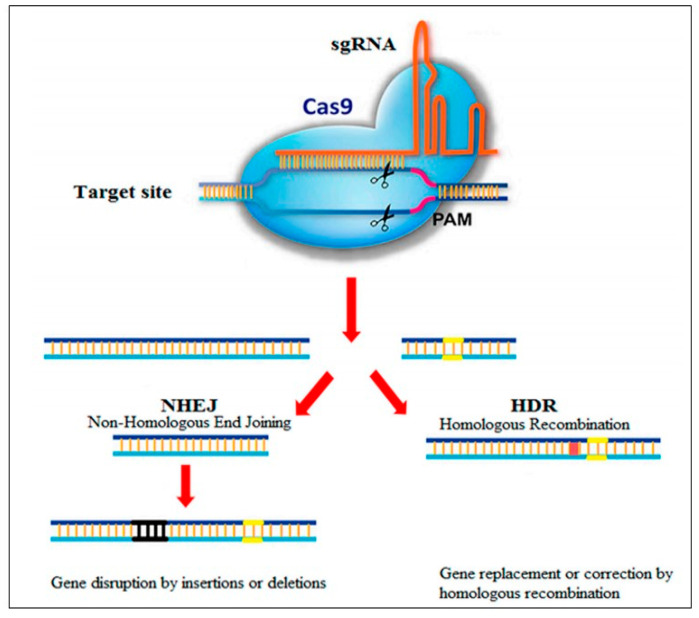
Schematic presentation of CRISPR-Cas9 genome editing system. The most widely used system in the field of genome editing is the type2 CRISPR-Cas9, which has three main components: (a) CRISPR RNA (cRNA), (b) the Cas9 endonuclease, and (c) a transactivating crRNA (tracr-RNA), which guides the Cas9 protein to its target and aids its function. The Cas9 protein cleaves the DNA, and the guide RNA targets the part of the DNA to be corrected. Adapted from Ref. [138].

CRISPR-Cas9 originated mainly from bacteria in which it is used as an immune defense system [134,135]. Bacteria infected by viruses seize small parts of viral DNA and bring them into the bacterial DNA, leading to the creation of segments known as CRISPR arrays. Using these CRISPR arrays, the bacteria “memorize” the viruses or the closely related ones, and if the bacteria are infected again by the same viruses, they generate RNA segments from the CRISPR arrays which identify and bind to specific regions of the viral DNA. The bacteria subsequently cleave the viral DNA using Cas9 or another similar enzyme and disable the virus. The bacterial CRISPR-Cas9 system has been adapted to edit human DNA. A small “guide” RNA binds a specific target sequence in a cell’s DNA in a way that brings to mind the bacterial process. This guide RNA also binds the Cas9 enzyme. The guide RNA after its introduction into cells identifies the target DNA sequence, and the Cas9 enzyme chops the DNA at the pre-specified location. Although Cas9 is the most commonly used enzyme, other enzymes are used depending on the desired targeted edits which include (i) conversion of DNA base pairs, (ii) deletion of DNA base pairs, (iii) insertion of DNA base pairs, or (iv) a combination of the above changes [136]. Once the target DNA is cleaved, the cell’s own DNA machinery is used to repair, add, or delete pieces of genetic material or to make changes to the DNA by replacing an existing segment with a customized DNA sequence (Figure 6) [137,138].

The NTLA-2001 is a gene-editing therapeutic agent specifically designed to treat ATTR amyloidosis by reducing the concentration of TTR in the serum that comprises a lipid nanoparticle encapsulating mRNA for Cas9 protein and a guide RNA-targeting TTR [139]. The NTLA-2001 was used for in vivo gene editing in six patients with ATTRv amyloidosis and polyneuropathy and significantly reduced the TTR serum level [140] with few and mild adverse events in the 28 days after infusion [140]. The potential benefit of NTLA-2001 in patients with ATTR- CA will be evaluated in a phase III, double-blind, randomized placebo-controlled trial [141].

The current DNA repair systems, however, have limitations as they differ in accuracy when establishing the appropriate genetic rectifications and may introduce unwanted mutations leading to disease. New techniques aiming at risk attenuation accompanied by gene editing are under development [142].

The siRNAs patisiran [31] and vutrisiran [143], as well as the ASOs inotersen [144] and eplontersen [145], have proved effective for the treatment of polyneuropathy caused by ATTRv amyloidosis in adults [146]. The advent of these disease-modifying agents has significantly reduced the role of liver transplantation for hTTR amyloidosis [147]. In the randomized APOLLO B trial, patients with ATTR-CA receiving patisiran exhibited significantly lower decline in functional capacity, as depicted by the 6 min walking distance, compared to placebo at 12 months of follow-up, without significant difference in the number of adverse events [148]. The recent randomized HELIOS-B trial showed that treatment with vutrisiran led to a lower risk of death from any cause and cardiovascular events than placebo and preserved functional capacity and quality of life in patients with ATTR-CA [149]. Interestingly, vutrisiran benefits were consistent regardless of background tafamidis therapy. A small, single-center, open label study demonstrated the efficacy (decrease in LV mass, improvement in functional capacity) and safety of inotersen in patients with hereditary or wild-type ATTR-CA [150]. The ongoing cardio TTRansform (NCT05667493), currently the largest randomized controlled study (≈1450 patients) in cardiac amyloidosis, will investigate the efficacy of eplontersen compared to placebo for the composite outcome of cardiovascular mortality and recurrent cardiovascular clinical events up to 140 weeks.

#### 4.2.2. TTR Stabilization

Tafamidis, a TTR stabilizer, is the only agent approved for the treatment of TTR amyloidosis as it reduces morbidity and prolongs survival, especially when it is given at an early disease stage, highlighting the importance of early diagnosis and treatment implementation [151,152]. *Acoramidis*, another TTR stabilizer binding a different site of TTR than tafamidis, did not reduce all-cause mortality [153].

#### 4.2.3. Anti-Amyloid Antibodies

Targeting myocardial TTR load is anticipated to result in restoration of cardiac function and prolonged patient survival. For this purpose, monoclonal antibodies (mABs) have recently been tested, and the initial results were encouraging [154,155].

### 4.3. Treatment of HF, Conduction Disturbances, and Arrhythmias

Diuretics are the mainstay of therapy for amyloid-related HF but must be used with caution due to the restrictive physiology that may be involved. Conventional HF medications are currently not widely prescribed in ATTR cardiomyopathy. However, retrospective studies suggest that low-dose β-blockers may reduce mortality risk in patients with an LVEF ≤ 40%, whereas mineralocorticoid receptor antagonists (MRAs) may be associated with reduced risk of mortality regardless of the LVEF [156]. Further, angiotensin-converting enzyme inhibitors (ACEi)/angiotensin receptor blockers (ARBs) may be safely used in severe cardiac phenotype, with a higher NYHA class and NAC disease stage, as well as in patients with chronic kidney disease stages [156,157]. Finally, it seems that SGLT-2i treatment in TTR cardiomyopathy patients is well tolerated and associated with favorable effects on HF symptoms, renal function, and a reduction in diuretic agent use. Further, SGLT2i reduced HF hospitalizations as well as cardiovascular and all-cause mortality, regardless of the LVEF [158]. The above findings are encouraging, but they should be confirmed in randomized control trials.

### 4.4. Heart Transplantation in Amyloid Cardiopathy

Amyloid CA, both in the setting of ATTR amyloidosis and AL amyloidosis, often progresses to end-stage HF and death. Traditionally, the results of heart transplantation have been considered worse in patients with amyloid cardiopathy compared with HF due to other causes, due to the nature of the disease affecting several systems [159]. However, there is recent evidence suggesting that with the current progress in amyloidosis treatment combined with an approach involving several disciplines and meticulous patient selection, the outcomes of patients with amyloid cardiopathy subjected to heart transplantation are encouraging [160,161].

## 5. Conclusions and Future Investigations

Promising diagnostic and therapeutic modalities are currently available for patients with cardiac amyloidosis. Prompt diagnosis and appropriate intervention have the potential to improve outcomes for these patients. Including amyloidosis in the differential diagnosis for patients evaluated for a variety of cardiac (e.g., restrictive cardiomyopathy, nonischemic dilated cardiomyopathy, and atrial fibrillation) and extra-cardiac (e.g., nephrotic syndrome, unexplained nonischemic cardiomyopathy, peripheral neuropathy, unexplained hepatomegaly, and atypical multiple myeloma) should improve diagnostic efficiency. Important unanswered issues in cardiac amyloidosis that remain and should be addressed include (a) differentiation between the biologic phenomenon of cardiac amyloid accumulation with aging and cardiac amyloidosis, (b) definition of the minimal disease burden justifying treatment implementation, and (c) non-invasive differentiation between ATTR-CA and AL-CA. Although genome editing has the potential to cure ATTRv amyloidosis by specifically modifying the mutated genes, several challenges still need to be fully addressed. Despite significant achievements in the field of amyloidosis, continued basic and clinical research efforts are needed to further improve quality of life and prolong the survival of patients with this disease.

## Figures and Tables

**Table 1:** Simplified diagnostic pathway for amyloidosis. Table compiled from Refs. [58,103].

Step 1	-Suspected amyloid cardiopathy (CA) (clinical, echocardiographic, magnetic resonance imaging “red flags”)
Step 2	-Exclusion of AL cardiopathy: determination of whether or not monoclonal free light chains (MFLC) are present in the serum or urine with immunofixation electrophoresis of the serum and urine and the serum free light chain (sFLC) assay, which measures circulating k and l free light chains and the k/l ratio.
Step 3	-Light chain amyloidosis (AL) excluded (no MFLC adjusted for eGFR and immunofixation abnormalities): cardiac scintigraphy with technetium-labeled radioactive tracers ATTR-CA. -AL not excluded (MFLC adjusted for eGFR and immunofixation abnormalities): hematology referral (fad aspirate, fat pad biopsy, bone morrow aspiration, and biopsy).
Step 4	-Cardiac scintigraphy: Peruggini score 0–1 excludes, whereas Peruggini score 2–4 confirms transthyretin amyloidosis CA (ATTR-CA). Hematology: If amyloid is present, AL-CA is confirmed.
Step 5	-ATTR-CA confirmed: transthyretin (TTR) sequencing should be performed to differentiate between ATTRwt (ATTR wild type) and ATTRv (ATTR variant). -AL-CA confirmed: amyloid typing (protein precursor identification).
Comment	Whenever suspicion of amyloidosis CA remains despite non-invasion investigation, cardiac biopsy should be performed. Depending on the availability and expertise, some centers will directly proceed with cardiac biopsy.

## Data Availability

Not applicable.
